# Cryo-EM Structure of a Possum Enterovirus

**DOI:** 10.3390/v14020318

**Published:** 2022-02-03

**Authors:** Ivy Wang, Sandeep K. Gupta, Guillaume Ems, Nadishka Jayawardena, Mike Strauss, Mihnea Bostina

**Affiliations:** 1 Department of Anatomy and Cell Biology, McGill University, Montreal, QC H3A 0C7, Canada; ivy.wang@mail.mcgill.ca; 2 AgResearch, Palmerston North 4442, New Zealand; sandeep.gupta@agresearch.co.nz; 3 Department of Microbiology and Immunology, University of Otago, Dunedin 9016, New Zealand; guillaume.ems@student.unamur.be (G.E.); gimshan.jayawardena@oist.jp (N.J.); 4 Faculté des Sciences, Université de Namur, 5000 Namur, Belgium; 5 Molecular Cryo-Electron Microscopy Unit, Okinawa Institute of Science and Technology Graduate University, Okinawa 904-0495, Japan; 6 Otago Micro and Nano Imaging, University of Otago, Dunedin 9016, New Zealand

**Keywords:** enterovirus, possum, capsid structure, cryo-electron microscopy

## Abstract

Enteroviruses (EVs) represent a substantial concern to global health. Here, we present the cryo-EM structure of a non-human enterovirus, EV-F4, isolated from the Australian brushtail possum to assess the structural diversity of these picornaviruses. The capsid structure, determined to ~3 Å resolution by single particle analysis, exhibits a largely smooth surface, similar to EV-F3 (formerly BEV-2). Although the cellular receptor is not known, the absence of charged residues on the outer surface of the canyon suggest a different receptor type than for EV-F3. Density for the pocket factor is clear, with the entrance to the pocket being smaller than for other enteroviruses.

## 1. Introduction

Enteroviruses (EVs) form the largest genus within the *Picornaviridae* family and are one of the most widespread viruses infecting animals. EVs are usually responsible for infections characterized by flu-like symptoms, but can be associated with more severe diseases following the spread of viral replication from the gastro-intestinal or respiratory tract to secondary tissues such as brain, heart, or liver [1]. Currently, there are 15 enteroviruses species that have been identified. EV-A to -D and Rhinoviruses (RV) RV-A to -C primarily infect humans, while EV-E to -L infect livestock and non-human primates [2,3]. Despite efforts, no antivirals for treating enterovirus infections are currently clinically approved [4,5]. With the constant characterisation of new species, EVs remain significant global health burden and increasing threat for further epidemics [6].

The picornavirus single stranded RNA genome encodes a single polyprotein that is cleaved into four structural proteins and seven non-structural proteins. During the viral lifecycle, the capsid precursor domain (P1) is cleaved into VP0, VP1 and VP3 which assemble into a protomer, the fundamental subunit capable of assembling into an icosahedral capsid. Following genome encapsidation, VP0 is further cleaved into VP2 and VP4 forming the mature capsid, with VP1–VP3 responsible for forming the outer surface, while VP4 lines the internal surface of the capsid. A large gallery of structures, solved initially by X-ray crystallography and more recently by cryo-electron microscopy (cryo-EM), have revealed a similar architecture among picornaviruses with distinct structural features [7,8]. A prominent star-shaped ‘mesa’ located at the five-fold axis is surrounded by a depression, named ‘canyon’, formed at the junction between VP1, VP2 and VP3. A pocket within VP1 containing a non-protein factor was identified in the majority of enterovirus structures. This “pocket factor” has been implicated in the regulation of viral uncoating. For many EVs, the canyon has been shown to be targeted by cellular receptors leading to the release of the pocket factor, destabilizing the capsid [9,10,11,12,13]. Alternatively, for EVs lacking a pocket factor, such as rhinovirus 14 (RV-14), the same effect can be obtained after exposure to low pH [14]. This leads to the externalization of membrane active peptides at the N terminal of VP1 and the release of VP4 followed by the release of the genome into the cytoplasm [15,16].

Currently, much of our knowledge of enterovirus structure and function comes from studies of human pathogens with limited study on non-human enteroviruses. Numerous EVs have been widely found in several species, posing a risk to global animal welfare [17]. Along with their high mutation rate and recombination frequency, these non-human EVs have the potential for cross-species infection and are therefore also of concern to human health [6,17]. Due to the close phylogenetic relationship between human and non-human picornaviruses, the designation of species is not based on the host species where it was originally identified. The EV taxonomy is continuously evolving with the addition of new species. Currently the species EV-E and EV-F are known to infect cattle [18,19], EV-G or porcine enteroviruses infect pigs, while simian EVs, commonly found in non-human primates, are classified into species EV-J and EV-H [17].

Structural studies on non-human enteroviruses are essential in understanding their conformational repertoire and identifying functionally important features for effective vaccine and novel anti-viral drug design. However, until now, structural information of non-human EVs was limited to the following bovine enteroviruses: EV-E1, also known as BEV-1 [12] and EV-F3, known as BEV-2 [20].

Here, we characterize the capsid structure from a possum enterovirus W6, classified as EV-F4, a recently isolated enterovirus from the Australian brushtail possum (*Trichosurus vulpecula*) [21]. Originally identified and characterized in an effort to improve biological possum management, this novel enterovirus provides a model that could provide insight into the structural basis of enterovirus properties that are common or differ between different enterovirus species. EV-F4 is closely related to EV-F3, suggesting that this may be the result of a cross-species infection that has adapted to grow in its possum host. We used cryo-EM to reconstruct the map of the EV-F4 capsid at a resolution of 2.96 Å and build an atomic model. The overall structure closely resembles EV-F3 but exhibits several differences in its capsid surface.

## 2. Materials and Methods

### 2.1. Virus Purification

Possum EV-F4 virus was cultured on primary possum kidney (PPK) cells according to the method described previously [21]. The PPK cells (passage 25–28) were maintained in complete Eagle’s minimum essential medium (cEMEM) containing 10% heat-inactivated foetal bovine serum, 0.1 M non-essential amino acids, 1 mM sodium pyruvate, 10 U/mL of penicillin, and 10 μg/mL streptomycin. EV-F4 (passage 6) virus stock was diluted 5-fold in cEMEM, and 10 mL of the diluted virus solution was transferred to the PPK cell monolayer grown in T175 flask and incubated at in CO_2_ incubator for 1 h. After the incubation, 15 mL fresh cEMEM was added to the flask and the cells were incubated at 37 °C in a humidified 5% CO_2_ incubator and observed for cytopathic effects under an inverted microscope. Once advanced cytopathic effects were visible, the virus was harvested by performing three cycles of freeze and thaw of the infected cells. Cell culture medium containing the virus was clarified by centrifugation for 20 min at 15,000× *g* at 4 °C and stored at −80 °C until used. The presence of the virus was confirmed by reverse-transcriptase PCR (data not shown) using the primer described previously [21].

To obtain a virus pellet, cell lysis supernatant was centrifugated for 2 h at 120,000× *g* at 4 °C by using a Beckman Coulter SW32Ti rotor mounted on Optima XPN-80 ultracentrifuge (Beckman Coulter, Brea, CA, USA). The virus pellet was resuspended overnight at 4 °C in caesium chloride (CsCl) purification buffer (137 mM NaCl, 5 mM KCl, 25 mM Tris base, 0.8 mM NaH_2_PO_4_, pH 7.4). Virus suspension was further clarified using a CsCl isopycnic gradient (1.33 g/mL) spinning at 61,580× g for 18 h at 22 °C in a SW32.1 Ti rotor mounted on Optima XPN-80 ultracentrifuge. Viral band was collected and dialysed against PBS overnight at 4 °C. Viral protein concentration in the purified fraction was quantified using Qubit protein concentration assay kit (catalogue #1814929; Life Technologies, Carlsbad, CA, USA).

### 2.2. Cryo-Electron Microscopy

An aliquot of 3.5 μL of purified EV-F4 full capsids at a concentration of 0.3 mg/mL was applied to Quantifoil R2/1 grids coated with 0.2 mg/mL of graphene oxide and previously glow discharged with a negative polarity at 15 mA for 15 s using a PELCO easiGlow. Samples were blotted with Whatman filter paper for 2.5 s before plunged into liquid ethane cooled with liquid nitrogen in a FEI Vitrobot MkIV. Vitrified samples were imaged using the FEI Titan Krios at 300 kV with a Gatan K3 Bioquantum camera and a nominal magnification of 105 kx. Movie frames were collected with a total dose of 41 e/Å^2^ over 30 frames with a final pixel size of 0.857 Å.

### 2.3. Image Processing

The EV-F4 full capsid electron potential map was reconstructed using single-particle processing in RELION (version 3.1) [7]. Movie frames were motion corrected using MotionCor2 [8] and CTF parameters were estimated using Gctf [9]. Particles were auto-picked using a template-free Laplacian-of-Gaussian filter-based procedure in RELION followed by discarding of bad particles based on 2D classification results. Selected particles underwent 3D classification imposing icosahedral (I1) symmetry and using the X-ray crystal structure of a related picornavirus (PDB accession number 3CJI) lowpass filtered to 50 Å as a reference map. Following 3D refinement with results from the 3D classification, sharpening was performed using RELION’s procedure for B-factor sharpening and calculating masked FSC curves to achieve a final resolution of 2.96 Å. All the figures were generated in UCSF ChimeraX [22].

### 2.4. Model Building

A preliminary model of the EV-F4 protomer in a mature virion was generated through modification of the X-ray crystal structure of EV-F3 (PDB accession number 5OSN) [5]. BLASTX sequence alignment with the nucleotide sequence of EV-F4 [4] and the protein sequence of EV-F3 [5] identified amino acid sequence differences between the two viruses. Amino acids in the EV-F3 molecular model were mutated using the Crystallographic Object-Oriented Toolkit [11] to correspond to the amino acid sequence of EV-F4. The preliminary EV-F4 protomer model was built in Isolde [23] and refined by Phenix [24].

## 3. Results and Discussion

**Figure 1:**
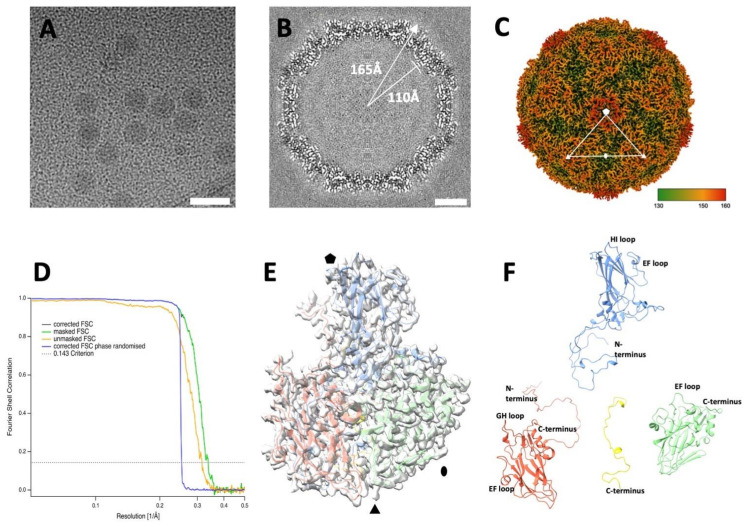
Cryo-EM reconstruction of the EV-F4 virion. (**A**) Representative micrograph of purified EV-F4 capsids. Scale bar, 50 nm. (**B**) Three-dimensional reconstruction central slice of the cryo-EM map. (**C**) Capsid surface electron potential map radially coloured according to the scale with red corresponding to a radius of 18 nm. The asymmetric unit was indicated by marking the five-, three-, and two-fold axes of symmetry with pentagon, a triangle, and an ellipse, respectively. (**D**) Resolution assessment of the cryo-EM reconstruction by Fourier shell correlation (FSC). (**E**) Atomic model of the protomer fitted into the cryo-EM density; (**F**) ‘Exploded’ view of atomic model of a protomer from the mature virion. Capsid proteins VP1, VP2, VP3 and VP4 are coloured in blue, green, red, and yellow, respectively.

EV-F4 virions were grown in PPK cells, purified via density gradient centrifugation, flash frozen and visualized by cryo-EM. Micrographs showed a population of homogeneous spherical particles with a diameter of ~35 nm (Figure 1A). After individual particle selection and single particle three-dimensional analysis, an electron potential map was reconstructed to a resolution estimated to be 2.96 Å (Table 1). The overall thickness of the spherical protein shell of the capsid is approximately 50 Å with an inner radius of 110 Å and outer radius of 165 Å (Figure 1B). The poorly ordered density within the capsid is consistent with the presence of an unordered RNA genome. The quality of the cryo-EM map enabled us to build an atomic model for EV-F4 for residues 10 to 274 of VP1, 9 to 244 of VP2, 1 to 243 to VP3, and 30 to 68 of VP4. The structure has the usual picornavirus architecture [7,8,25,26,27], with the three major capsid proteins having a similar β-barrel fold with the following two β-sheets, each formed by four β-strands: BIDG and CHEF, while VP4 is located along the interior of the capsid.

Consistent with the close evolutionary relationship between EV-F3 and EV-F4, their capsid proteins show remarkable sequence and structural similarities, as follows: the sequence similarities of VP1, VP2, VP3, and VP4 between EV-F4 and EV-F3 are 84%, 91%, 91%, and 93%, respectively, while between EV-F4 and EV-E1 are 54%, 75%, 64%, and 76%, respectively. Correspondingly, the superimposition of the atomic model of the EV-F4 protomer and the EV-F3 protomer shows an overall root-mean-square deviation (RMSD) of atomic positions of 0.76 Å, while a comparison with EV-E1 shows a RMSD of 0.63 Å. The interior of the EV-F4 capsid is similar to EV-F3 and EV-E1 capsids. VP4 is structurally almost identical in EV-F4, EV-F3 but shorter than in other EVs where the N-terminal region stretches from the 5-fold axis towards the 3-fold axis.

**Figure 2:**
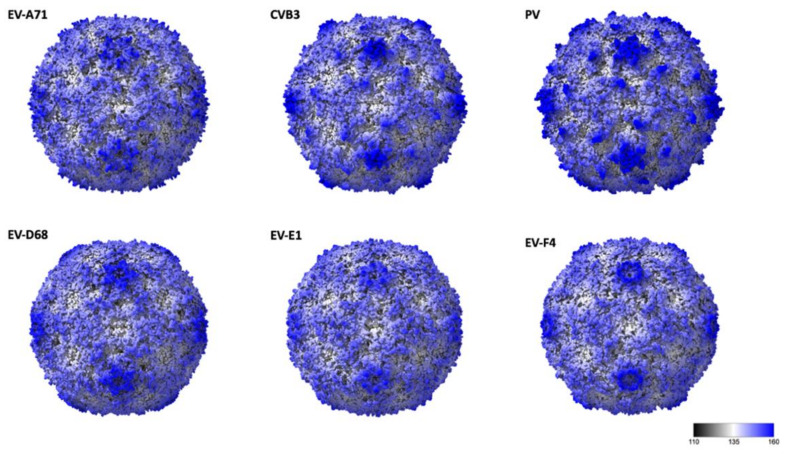
Radially coloured surface representations of enteroviruses from different species: EV-A71 (3vbs), Coxsackie virus B4, VB3 (1pov), Poliovirus (1hxs), EV-D68 (6csg), EV-E1 (1bev), and EV-F4. Scale corresponds from black to blue corresponding for a radius of 11 nm and 18 nm, respectively.

The general arrangement of the 180 β-barrel cores corresponding to the icosahedral copies of VP1, VP2 and VP3 is similar to other EV structures, while differences in surface loops are responsible for the distinct external features of different EVs [7,8,28,29]. EV-F4 loops, as for EV-E1 and EV-F3, are shorter giving the capsid a much smoother appearance (Figure 2). In poliovirus, the BC loop of VP1 protrudes to form the arm of a star-like mesa, while the DE loop extends inwards to fill in the centre of the mesa. The same loops responsible for the prominent five-fold mesa in poliovirus or EV-D68 are shorter in EV-E1, EV-F3, and EV-F4 (Figure 2). Shortening of VP1 BC and DE loops in EV-F4 and EV-F3, but also in EV-A71 results in circular 5-fold mesas that remains open in the middle instead of the occluded star-shaped mesas found in poliovirus. The tips of the propellers located around the 3-fold axis characteristic of other EVs are absent in EV-F4 and EV-F3 (Figure 1C and Figure 2).

**Figure 3:**
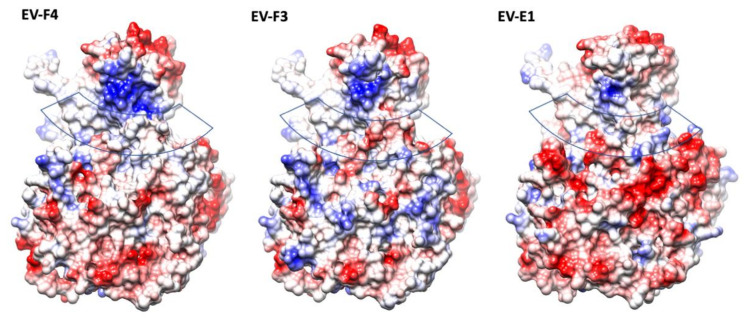
The electrostatic surface representation of protomers from structures of animal EVs; the surfaces are coloured in gradient from blue to red corresponding from positive to negative charge, from 10 to −10 kcal/mol·e respectively. The approximate location of the canyon is indicated by a transparent band.

The presence of a canyon depression in EVs has been shown to play an essential role in the interaction with the cellular receptors, as proven by numerous studies [10,28,29,30]. With a geometry which makes them a difficult target for antibodies, canyons are a prominent structural feature in EVs. Different geometries of the VP1 BC and GH loops, and VP2 EF loops dictate the interaction with their cellular receptors [29]. In both EV-F4 and EV-F3 the VP3 GH loop insertion extends into the canyon between the tip of the 3-fold propellers and the 5-fold mesas (Figure 1). Additionally, there is an outward shift in the C terminal bend in VP1 and the C terminal of VP3 extending along the floor of the canyon. The exact function of the VP3 GH loop is unclear, but the equivalent loop in cardioviruses is required for infection [31], suggesting that it could be involved in the attachment to the receptor [12]. While little is known regarding EV-E and -F receptors or antigenicity, a shallower canyon suggests improved cellular receptor accessibility and reduced immunological seclusion. The EV-E and -F overall canyon geometry is similar; however, the electrostatic landscape of the capsid is different when comparing EV-F4 to existing structures. EV-F4 does not display any charged residues in the canyon region, while the corresponding region in EV-F3 is slightly negatively charged (Figure 3). EV-E1 also has a broadly neutrally charged canyon, but with a cluster of strongly negatively charged residues on the south wall.

Interestingly, in EV-F4, the north rim of the canyon has a prominent patch of positively charged residues, which is reduced in EV-F3 and further reduced in EV-E1. The top of the mesa has a similar negative charge distribution in all the EV-E and -F capsids, whereas around the 3-fold axis the EV-F4 capsid displays a gallery of negative residues more similar to EV-E1, while EV-F3 has a mosaic of negative and positive charges.

**Figure 4:**
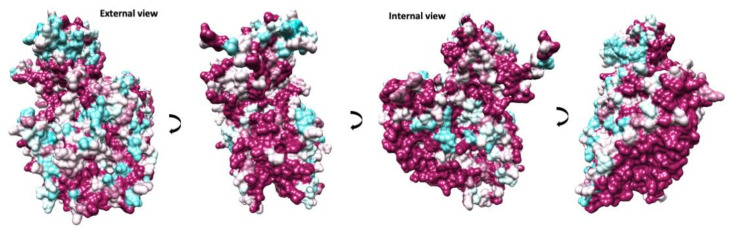
Conservation of protomer in animal EV capsids. Different views are indicated by 90-degree rotations starting with an external view perpendicular to the capsid surface. The protomer surface is coloured in a gradient from cyan to maroon according to sequence conservation from low to high, respectively.

A comparison between available EV-E and -F sequences reveals that the interface between protomers assembled in a mature capsid is very well conserved (Figure 4). Two α-helices located within VP2 from adjacent protomers at the two-fold axis have been demonstrated to move apart in other enteroviruses to open up a channel and suggested to interact with the RNA genome [16,32]. In EV-E and -F, there is a similar arrangement of VP2 α-helices (residues 88 to 96) with residues involved in the contact very well conserved. On the contrary, major amino acid differences are present at the exterior of the capsid, with few conserved regions between the existing isolates, dictating different surface properties. Indeed, it was shown that common VP2 linear epitopes are shared between EV-E and -F isolates, but these are situated either in the interior or at the interface with VP3 [33]. It was reported that in the case of EV-E1, several internal residues are involved in contact with special viral genome sequences acting as packaging signals [14,34,35]. For instance, using asymmetrical reconstruction, in the proximity of a conserved Trp residue in VP2, ordered density was assigned to EV-E1 viral RNA [34]. We attempted a non-icosahedral reconstruction and focused classification, but we were not able to unambiguously confirm the presence of ordered RNA loops. However, the interior of the capsid in the region adjacent to Trp36 is not particularly well conserved between EV-E and -F viruses (Figure 4).

**Figure 5:**
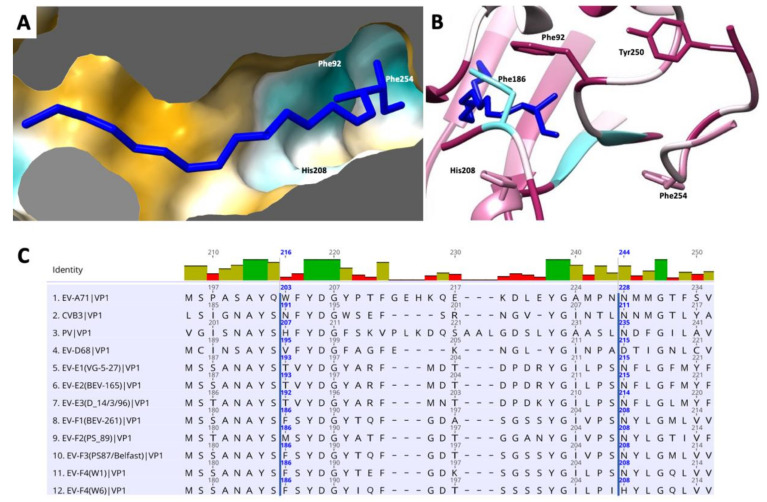
Representation of the hydrophobic pocket on EV-F4 mature particle. (**A**) Surface representation coloured in a gradient with golden and cyan areas denote the most hydrophobic and hydrophilic areas, respectively. (**B**) Entrance into pocket factor with ribbon representation coloured from cyan to maroon according to sequence conservation animal EVs, from low to high, respectively; several residues guarding the entrance into the pocket are indicated. (**C**) Sequence alignment of residues guarding the hydrophobic pocket in several enteroviruses. EF-F4 is last in this list.

At the bottom of the canyon lies the entrance to a hydrophobic pocket that starts at the base of the VP1 β-barrel and extends toward the five-fold axis. As in the majority of enteroviruses, this cavity harbors a ‘pocket factor’—a fatty acid ligand—that has been implicated in the stabilization of the capsid and in the regulation of enterovirus uncoating during infection. We modelled this fatty acid as a sphingosine, similar to the models of EV-F3 and EV71 [36]. The pocket is lined with hydrophobic residues that are highly conserved between EV-E and EV-F species (Figure 5). The release of the pocket factor from the VP1 pocket has been shown to be required for the transition from the mature capsid into an expanded state necessary for uncoating [37]. The stability of the mature particle is determined in part by the stabilizing interactions between the pocket factor head group and residues that form the entrance. Similar to other enteroviruses, in EV-F4, the pocket entrance is formed by part of the CD loop (residues 90 to 93), GH loop (residues 204 to 210) and the C terminal (residues 253*–*255). In both EV-F3 and EV-F4, the pocket entrance is reduced in size by bulky side chains from residues 209 and 254. Near the head group of the pocket factor the Phe186 contributes to this geometry. The lipid head group is stabilized by hydrogen bonds formed with the main chain and residues surrounding the entrance to the pocket [12,38]. In EV-F4, His208 replaces Asn208 in EV-F3 and -E1 as well as in the majority of EVs, introducing a charged residue in proximity to the head of the fatty acid pocket factor. Asn208 is responsible for forming stabilizing hydrogen bonds with the [carboxyl/hydroxyl] lipid head group, while the presence of His208 increases favourable van der Waals contacts between H208 and the lipid head group (Figure 5B). Additionally, differences in EV-F4 pocket entrance residues leads to a reduction in hydrogen bonding in the pocket entrance. These changes in the pocket entrance properties suggests different pocket factor dynamics and consequently capsid stability between EV-F4 and other related enteroviruses.

## 4. Conclusions

Picornaviruses are ubiquitous in animals worldwide, posing a constant danger to human and animal health. While foot-and-mouth disease virus is the major threat to the farming industry, other species have the potential to expand their species tropism or to evolve toward higher virulence. Enteroviruses are commonly found in diverse domesticated species [18] with bovine viruses the most well characterized [19,39]. Of these, numerous isolates have been classified into the following two genera: enterovirus E with four identified serotypes of enterovirus isolated from cattle, and enterovirus F species with five virus serotypes identified in cattle [40]. A sixth serotype from a brushtail possum (*Trichosurus vulpecula*) was reported in New Zealand [21], where it is an invasive species. Novel viruses are usually described in domestic animals, leaving unanswered the origin of their natural reservoir. A better understanding of enterovirus diversity at a structural level can help to explain viral phylogeny and inform future vaccine design. Currently, EV-F4 infection in Australian brushtail possums has not been observed to cause any disease phenotype. These viral species are commonly found not only in animals exhibiting clinical signs, but also in healthy animals.

We have reconstructed a cryo-EM map of the EV-F4 and built the atomic model for the capsid proteins. Overall, EV-F4 shows great similarity with bovine enteroviruses EV-F3 and EV-E1. Together, they show morphological characteristics distinct from enteroviruses infecting humans. Although the differences in the chemical properties of residues between EV-F3 and EV-F4 has minimal effects on overall structure, this leads to differences in the electrostatic and hydrophobic properties of the 5-fold mesas, canyons and VP1 pocket that potentially mediate species tropism and affect infectivity. Structural characterization animal EVs can identify conserved regions and inform rational anti-viral development and vaccine design against this increasingly prevalent genus of viruses.

## Figures and Tables

**Table 1:** Summary of cryo-EM data collection, processing, and refinement statistics.

Parameter	Value
Data collection	
Microscope	FEI Titan Krios
Voltage (kV)	300 kV
Magnification	105000
Detector	Gatan K3 Bioquantum
Pixel size (Å/pixel)	0.857
Total electron dose	41 e^−^/Å^2^
Number of Frames	30
Defocus range (μm)	0.5–3.5
Motion correction	MotionCor2
Micrographs images (no.)	2445
3D reconstruction	
Software	Relion-3.1
Final number of particles in the reconstruction	9687
Imposed symmetry	I1
Fourier shell correlation (FSC) cut-off	0.143
Map resolution (Å)	2.96
Atomic modelling and refinement statistics	
Software	COOT, ISOLDE, Phenix
Homology model (PDB accession code)	5OSN
B-factor (Å^2^)	−100
Total number of atoms	6570
Protein atoms	6344
Other atoms	226
RMSD^2^ bond length (Å)	0.002
RMSD^2^ bond angles (^o^)	0.458
Molprobity score	0.88
All atom clashscore	1.43
Cβ deviations	0
Ramachandran plot (%)	
Favoured Allowed Outliers	98.41 1.46 0.13

## Data Availability

The capsid was deposited in the Electron Microscopy Data Bank (EMDB) with accession numbers EMD- 25905 and the corresponding model as PDB entry 7thx.
